# Circulating sex hormones, specifically estrogen in females and testosterone in males, are associated with ambient environmental temperature during prolonged physical training

**DOI:** 10.1080/23328940.2026.2710566

**Published:** 2026-08-09

**Authors:** Gabrielle E. W. Giersch, Karleigh E. Bradbury, Brittany Bozzini, Katelyn Guerriere Aaron, Leila A. Walker, Stephen Foulis, Julie M. Hughes, Nisha Charkoudian, Kristin L. Popp

**Affiliations:** a US Army Research Institute of Environmental Medicine, Natick, MA, USA; b Oak Ridge Institute for Science and Education, Belcamp, MD, USA

**Keywords:** Reproductive health, ambient temperature, exercise training, urinary hormones, military health

## Abstract

Circulating sex hormones play important roles in reproductive and non-reproductive physiological homeostasis. We evaluated the relationships of sex hormones measured in urine and serum with ambient temperature (maximum and average daily temperatures) in male and female soldiers undergoing initial military training (*n* = 237, 125F; data are mean ± SD; age: 21 ± 4 years, BMI: 24 ± 3, body fat: 28 ± 7%). Participants provided intermittent blood samples for assessment of 17β-estradiol, total and free-testosterone, luteinizing hormone (LH), and sex hormone binding globulin. Females not using hormonal contraceptives (non-HC users) additionally provided daily first morning urine samples which were analyzed for metabolites of estrogen normalized to creatinine (estrone conjugates, E1C) and progesterone (pregnanediol glucuronide, PDG), LH, and follicle stimulating hormone (FSH). Linear mixed effects models were used to evaluate the relationship between maximum and average daily temperatures and circulating hormonal and gonadotropin concentrations. In urine collected from female non-HC users, maximum daily temperature was inversely associated with E1C and FSH (*p* = 0.0019, *p* < 0.001, respectively). Total testosterone was inversely related to maximum daily temperature in males (*p* < 0.001) and positively associated in both groups of females (non-HC users: *p* = 0.004, HC users: *p* = 0.018). Estradiol was inversely related to maximum daily temperature in female non-HC users only (*p* = 0.001). There was no relationship between temperature (maximum or average) and serum or urine LH, or urine PDG. These findings suggest that ambient temperature is associated with modest sex- and hormone-specific variation in circulating sex hormone concentrations during prolonged physical training.

## Introduction

It is well established that reproductive dysfunction and sex steroid hormonal dysregulation can result from stressors such as physical activity, energy imbalance [1,2], psychological stress [3,4], and lack of sleep [5,6]. Environmental factors like ambient temperature are not usually considered as one of the stressors influencing reproductive health in humans, despite extensive animal research describing that relationship [7]. Specifically, in animals, high environmental temperatures appeared to reduce fertility and blunt sex steroid hormones in murine and bovine models when housed outside [8–10]. Similarly, in birds and small mammals, heat stress was associated with reproductive/estrous cycle disturbances [11–13].

However, few studies have evaluated the impact of ambient temperature on human reproductive health. Bull et al. [14] reported that ambient temperature did not influence length of menstrual cycle in premenopausal women. However, higher ambient temperatures have been shown to be inversely associated with ovarian reserve [15] highlighting that ambient temperature may be a contributor to reproductive health in humans. This highlights the need to evaluate how environmental temperatures may compound additional stressors often experienced by personnel in physically demanding occupations.

Extensive physical training, particularly when combined with additional stressors such as inadequate energy availability, psychological stress, and insufficient sleep, can be a detriment to reproductive health by suppressing the hypothalamic-pituitary-gonadal axis [16–18]. This is thought to result from inhibition of sex hormone production and release [19]. Prolonged and intense physical activity in the absence of adequate recovery can lead to adverse reproductive health outcomes, including anovulatory menstrual cycles and poor bone health in males and females [18,20–23]. These stressors commonly co-occur in physically active and military populations, complicating efforts to isolate the contribution of any single exposure.

Strenuous and prolonged periods of physical activity are common during initial military training scenarios as well as during specific elite-level training – such as the US Army Rangers, US Navy Seals, etc., and during deployment. These military environments can lead to multi-system physiological perturbations, including endocrine dysregulation, altered iron homeostasis and suppressed immune function [24]. We recently reported discordant data between self-reported menstrual function and discrete measurement of menstrual health. Among women undergoing US Army Basic Combat Training (BCT), 42% reported normal menstrual cycles during BCT, but over 85% showed no evidence of ovulation and/or short luteal phases, suggesting subclinical menstrual dysfunction [25]. Subclinical menstrual dysfunction refers to a myriad of disorders ranging from inadequate concentrations of sex hormones to anovulation [26,27]. Additional research has shown prevalence of subclinical menstrual disorders as high as 46% in physically active populations [28]. In males participating in US Army Ranger training, previous research has shown multifactorial stressors (intense training, sleep deprivation) prompt reductions in circulating testosterone concentration [29].

Additionally, military training is often conducted in hot environments, with previous reviews highlighting the influence of outdoor training on health in a military population [30–32]. The threats of heat stress are often associated with the risk of heat illness [33,34], physiological impacts (e.g. dehydration, acute kidney injury) and performance detriments. It is unclear whether heat stress during initial military training contributes to variability in reproductive hormone levels beyond the effects of other concurrent stressors.

The purpose of this exploratory analysis was to evaluate the relationship between ambient temperature (maximum and average daily temperatures) and circulating levels of reproductive hormones observed during periods of initial military training in young healthy women and men. We also evaluated the relationship between training time (both week and day) and sex hormone concentrations. We hypothesized that elevated environmental temperatures would be associated with lower levels of sex hormones and that training time would be associated with increases in sex hormones (i.e. testosterone).

## Methods

This analysis was part of a larger investigation evaluating musculoskeletal injury risk during BCT in male and female volunteers. These data represent a secondary analysis of a unique research question from that data set, with previous results reported elsewhere [25,35,36]. These data were collected between August and October 2021 at Fort Jackson in Columbia, South Carolina, USA. In brief, the investigators have adhered to the policies for protection of human participants as prescribed in Department of Defense Instruction 3216.02, and the research was conducted in adherence with the provisions of 32 Code of Federal Regulations Part 219, where all volunteers provided written and informed consent, and the protocol was approved by the institutional review board at the Medical Research and Development Command (protocol no. M-10678). This investigation conformed to all aspects of the *Declaration of Helsinki*, apart from being registered in a database.

Data collection took place over the course of 10 weeks of BCT in US Army Soldiers. Participants were asked to fill out detailed questionnaires on their personal and health history, including location previously lived prior to arrival to Columbia, SC, and amount of time lived at that location, and females completed additional questions relating to menstrual/reproductive health, including menstrual regularity and current contraceptive use. These questionnaires were utilized to determine volunteers who would provide daily urine samples. Female Soldiers who were not currently using hormonal contraceptives (non-HC users), as determined by self-reporting, provided daily first morning urine samples for the analysis of estradiol (estrone conjugates, E1C), luteinizing hormone (LH), follicle stimulating hormone (FSH), and the progesterone metabolite pregnanediol glucuronide (PDG). All urinary biomarkers were analyzed using competitive immunoassay (Centaur XP, Siemens Healthcare Diagnostics, Erlangen, Germany, sensitivity < 0.05 µg·mL^−1^) and corrected for creatinine concentration in the urine. Inter‑ and intra-assay coefficients of variation, respectively, are as follows: (E1C: 11.5, 8.1%; PDG: 17.8, 7.7%; FSH: 6.6, 5.0 %; LH: 4.8, 3.4%). All participants and groups (male, female hormonal contraceptives users and female non-contraceptive users) provided fasted and rested blood samples in the morning between 5:00 am and 8:00 am at the following time points: Week 1 (Pre), Week 2, Week 4, Week 6, Week 8, and Week 9 (Post). Serum and plasma were centrifuged, aliquoted, frozen, and shipped to Brigham Research Assay Core (Boston, MA, USA) to evaluate free- and total-testosterone, 17β-estradiol, LH, and sex-hormone binding globulin (SHBG). Solid-phase extraction, high-performance liquid chromatography, and liquid chromatography tandem mass spectrometry (LC-MS/MS) with electrospray ionization were used to quantify total testosterone with intra-assay CVs < 5% and inter-assay CVs < 7%. LC-MS/MS was also used to quantify 17β-estradiol with intra-assay CV of <5% and inter-assay CV < 12%. LH and SHBG were quantified using chemiluminescent immunoassay with intra-assay CVs ranging between 4.3–6.7% and inter-assay CVs ranging between 4.3–7.9%. Samples from each individual were measured within the same assay batch to reduce inter-assay variability.

Maximal, average, and minimum daily ambient temperature (T_amb_) data was retrieved from the closest weather station of the National Weather Service with daily temperature data (Columbia Metropolitan Airport, SC Automated Surface Observing System/Automated Weather Observing System – Columbia Metropolitan Airport, Elevation 71 m, Latitude/Longitude: 33.94194/-81.11806). Daily temperatures from the day before (day t-1) measures of blood and urine sex hormones were utilized as samples were collected in the morning, prior to any daytime high temperatures. Locations previously lived were characterized into geographical regions based on similar climate per the National Oceanic and Atmospheric Administration (NOAA; https://www.ncei.noaa.gov/access/monitoring/reference-maps/us-climate-regions). All volunteers had lived in the respective previous location for at least 6 months as evaluated via entry questionnaire.

### Statistical analyses

As this was an exploratory analysis, an *a priori* power calculation was not performed. One-way analysis of variance with *Bonferroni* post hoc corrections was utilized to evaluate differences in anthropometric characteristics between groups. Linear mixed-effects models were used to evaluate the relationship and relative influence of maximal and average ambient temperatures on hormonal concentrations collected from daily urine and intermittent blood samples. Day and week of training, for urine and blood, respectively, were included in the model to account for training and to evaluate how the hormones changed over time. To account for non-normally distributed outcomes and non-normally distributed residuals, standard errors were bootstrapped with 100 replications to ensure valid inference using the regression coefficient *p*-values and accuracy of hypothesis testing. Location was included in the model to account for regional differences prior to beginning BCT. Group based on sex and contraceptive use (i.e. males, female non-HC users, female HC users) was also included in the model and volunteer number was included as the random factor in each linear mixed-effects model. Post hoc comparisons were conducted to evaluate differences between, controlling for sex, and the interaction between group and temperature (both maximum and average). All statistical analyses were conducted using Stata (Stata SE 19, Statacorp, College Station, TX). Significance level was set *a priori* at *p* ≤ 0.05.

## Results

Anthropometric information is found in Table 1 (*n* = 237). Males were taller, heavier, and had a higher BMI relative to their female counterparts in both groups. Females in both groups had greater body fat percentage than males. There was no difference between the two groups of females in anthropometric characteristics and no difference in age among the 3 groups (*p* > 0.05). Sixty-two female (59%) non-contraceptive users provided daily urine samples.

**Table 1.** Anthropometric characteristics for volunteers. Data are represented as mean ± SD; cm = centimeters, kg = kilograms, BMI = body mass index. * denotes (*p* < 0.05) compared to males, and ** denotes (*p* < 0.01) compared to males. One‑way analysis of variance with *Bonferroni* post hoc corrections was utilized to evaluate differences in anthropometrics between groups. Previous locations, based on NOAA pre-defined geographical areas, are shown in the lower panel. Percentages are relative to each group (i.e. non-HC users, HC-users, males, and total).

	Female (*n* = 125)			
	non-HC (*n* = 105)	HC users (*n* = 20)	Male (*n* = 112)	Total (*n* = 237)
Age (years)	21 ± 5	20 ± 4	21 ± 3	21 ± 4
Height (cm)	161.3 ± 6.3**	162.9 ± 5.5**	175.1 ± 8.0	168.1 ± 9.5
Weight (kg)	62.2 ± 8.2**	61.3 ± 7.8**	78.0. ± 13.5	69.2 ± 13.5
BMI	24 ± 3*	23 ± 3**	25 ± 4	24 ± 3
Bodyfat (%)	31.6 ± 5.5**	31.1 ± 5.8**	23.5 ± 7.0	27.8 ± 7.4
Location				
	non-HC	HC-users	Males	Total
Northeast US	n = 1 (0.1%)	n = 2 (10%)	n = 2 (1.8%)	n = 5 (2.1%)
Ohio Valley	n = 9 (8.5%)	n = 2 (10%)	n = 11 (9.8%)	n = 22 (9.3%)
Southeast	n = 39 (37.1%)	n = 5 (25%)	n = 37 (33.0%)	n = 81 (34.1%)
Upper Midwest	n = 8 (7.6%)	n = 1 (5%)	n = 15 (13.4%)	n = 24 (10.1%)
South	n = 15 (14.3%)	n = 3 (15%)	n = 18 (16.1%)	n = 36 (15.1%)
Southwest	n = 2 (1.9%)	n = 1 (5%)	n = 5 (4.5%)	n = 8 (3.3%)
Northwest	n = 3 (2.9%)	n = 1 (5%)	n = 3 (2.7%)	n = 7 (3.0%)
West	n = 9 (8.6%)	n = 1 (5%)	n = 10 (8.9%)	n = 20 (8.4%)
Northern Rockies	n = 1 (1%)	n = 0	n = 0	n = 1 (0.4%)
Other – Caribbean	n = 1 (1%)	n = 0	n = 3 (2.7%)	n = 4 (2.1%) −1
Other – Europe/Asia	n = 5 (4.8%)	n = 1 (5%)	n = 1 (0.9%)	n = 7 (3.0%)
Other – Africa	n = 2 (1.9%)	n = 0	n = 0	n = 2 (0.9%)
Other – Unknown	n = 10 (9.5%)	n = 3 (15%)	n = 7 (6.3%)	n = 20 (4.2%)

### Urinary sex hormones

**Figure 1.**
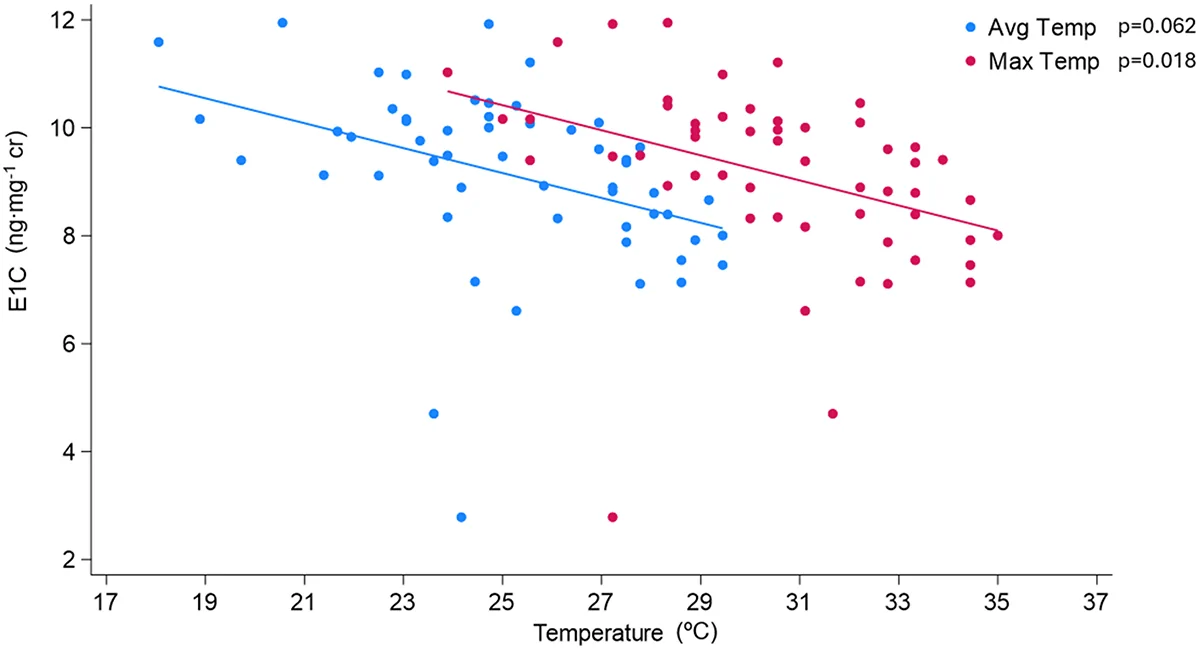
The relationship between the maximum and average daily temperatures and E1C in female non-contraceptive users from daily urine samples. Linear mixed‑effects models show a significant relationship between maximum temperature (*p* = 0.018) and average temperature (*p* = 0.062) and E1C values. Data presented are mean values for each temperature.

**Figure 2.**
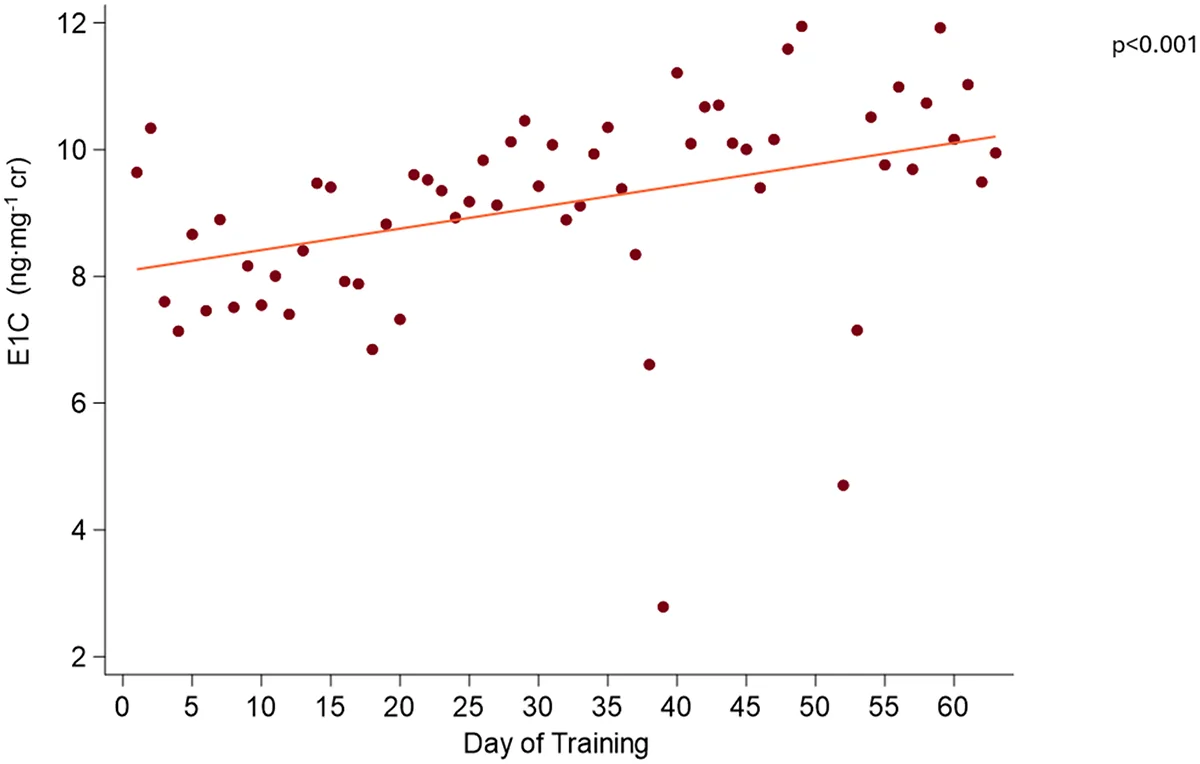
Relationship between day of training and E1C in female non-contraceptive users from daily urine samples. Linear mixed‑effects models show a significant relationship between day and E1C values (*p* < 0.001). Data presented are mean values for each temperature.

**Figure 3.**
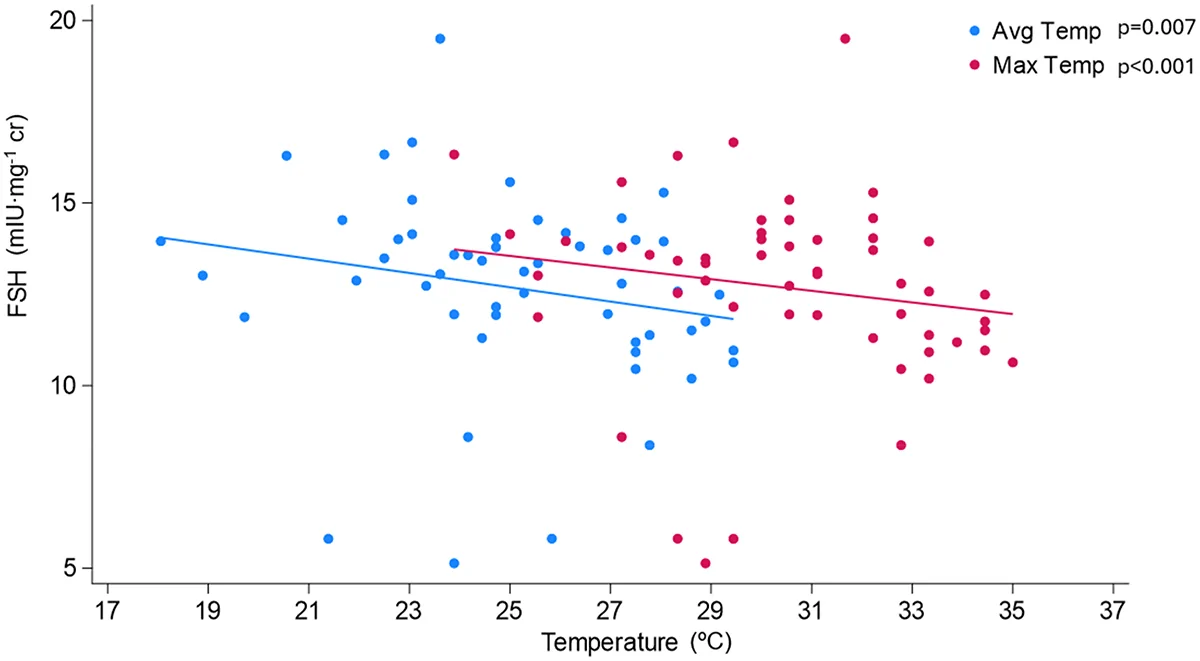
The relationship between the mean maximum and mean average daily temperatures and FSH in female non-contraceptive users from daily urine samples. Linear mixed‑effects models show a significant relationship between maximum temperature (*p* < 0.001), average temperature (*p* = 0.007) and FSH values. Data presented are mean values for each temperature.

Urinary E1C was negatively associated with maximum daily temperature (*p* = 0.018) but the relationship with average temperature did not reach statistical significance (*p* = 0.062; Figure 1, Table 2). Day of training was positively associated with E1C such that as days of training increased, E1C increased (*p* < 0.001; Figure 2, Table 2). FSH was negatively associated with maximum (*p* < 0.001) and average temperatures (*p* = 0.007; Figure 3, Table 2). LH and PDG were unrelated to any temperature values (all *p* > 0.05). FSH, LH and PDG were unrelated to day of training (*p* > 0.05).

**Table 2.** Linear mixed-effects model results evaluating the relationship between maximum and average daily temperatures in E1G, FSH, LH, and PDF from daily urine samples in female non-HC users (*n* = 62). (t-1) refers to the maximal or average daily temperature from the day before the hormonal measurement.

Panel A	E1C				FSH			
	*β ± SE*	*p*	*β ± SE*	*p*	*β ± SE*	*p*	*β ± SE*	*p*
Max Temp (t-1)	**-0.131 ± 0.052**	**0.011**			**-0.313 ± 0.082**	**0.000**		
Day	**0.036 ± 0.008**	**0.000**			0.000 ± 0.013	0.994		
Avg Temp (t-1)			−0.107 ± 0.057	0.062			**-0.216 ± 0.080**	**0.007**
Day			**0.039 ± 0.009**	**0.000**			0.010 ± 0.014	0.451
Intercept	13.650 ± 1.730	0.000	12.271 ± 1.645	0.000	32.619 ± 3.407	0.000	28.206 ± 2.951	0.000
N	2,315		2,315		2,344		2,344	
	**LH**				**PDG**			
**Panel B**	***β ± SE***	***p***	***β ± SE***	***p***	***β ± SE***	***p***	***β ± SE***	***p***
Max Temp (t-1)	−0.029 ± 0.042	0.487			0.006 ± 0.004	0.202		
Day	−0.008 ± 0.006	0.135			−0.001 ± 0.001	0.296		
Avg Temp (t-1)			−0.054 ± 0.048	0.256			0.002 ± 0.004	0.576
Day			−0.011 ± 0.006	0.051			−0.001 ± 0.001	0.122
Intercept	4.400 ± 1.428	0.002	4.953 ± 1.381	0.000	0.135 ± 0.148	0.361	0.261 ± 0.120	0.029
N	2,302		2,302		2,385		2,385	

Each column contains results from a separate linear mixed effects model which controls for a fixed effect for location.

### Circulating sex hormones

**Figure 4.**
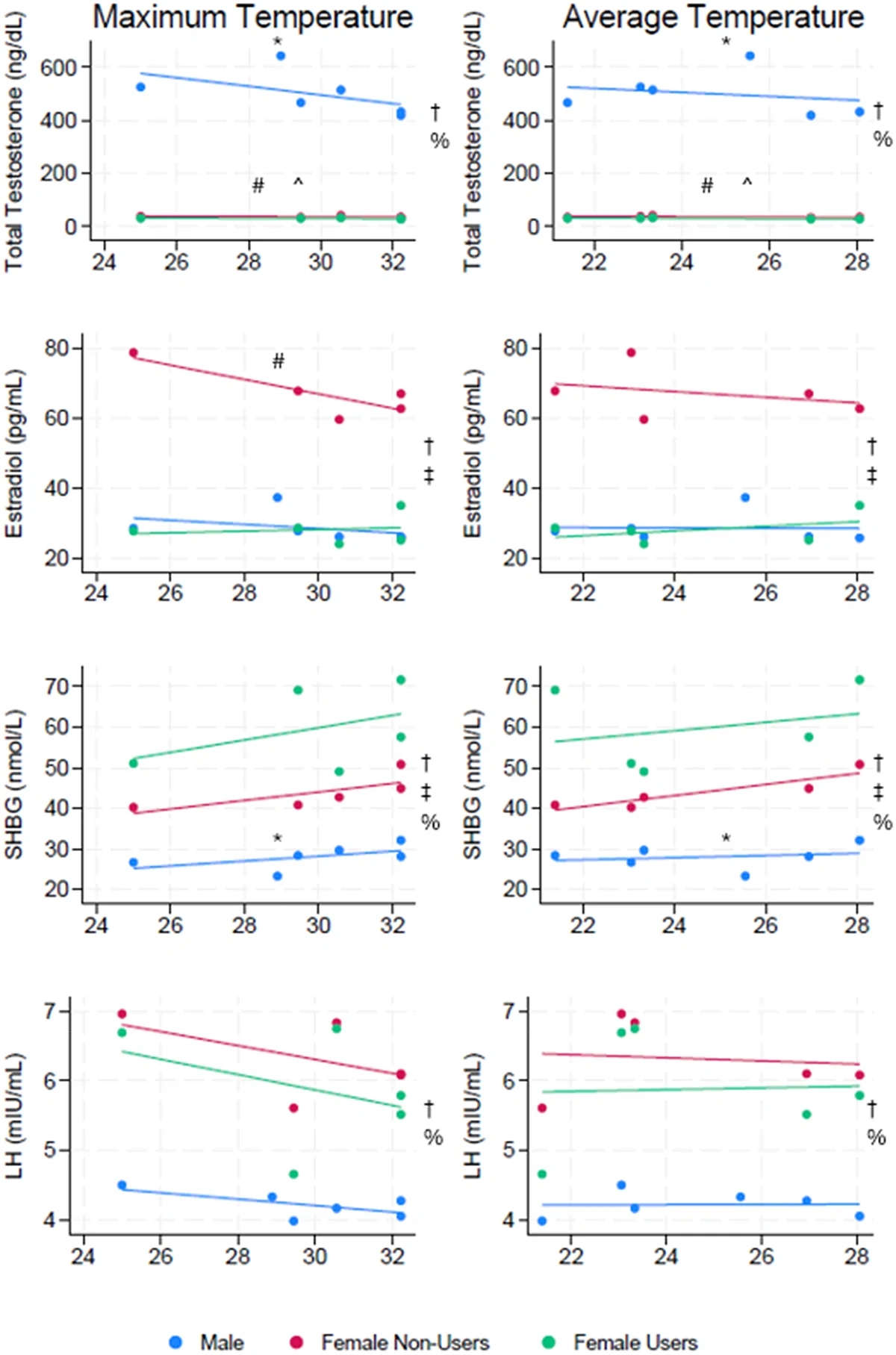
Relationship between blood hormones and maximal and average daily temperatures in males, shown in blue (*n* = 112), female non-contraceptive users, shown in red (*n* = 62), and females contraceptive users, shown in green (*n* = 63) via *posthoc* pairwise comparisons following the linear mixed‑effects model. 3A and 3B. Relationship between testosterone and maximal and average daily temperatures, respectively. 3C and 3D. Relationship between estradiol and maximal and average daily temperatures, respectively. 3E and 3F. Relationship between SHBG and maximal and average daily temperatures, respectively. * denotes a statistically significant change over the temperature range in males (*p* < 0.05), # denotes a statistically significant change over the temperature range in female non-contraceptive users (*p* < 0.05), ^ denotes a statistically significant change over the temperature range in female hormonal contraceptive users (*p* < 0.05), † denotes a difference between males and female non-contraceptive users, ‡ denotes a difference between female non-contraceptive users and female hormonal contraceptive users, and % denotes a difference between female hormonal contraceptive users and males.

Serum total testosterone was negatively related to maximal daily temperature in males (*p* < 0.001) and positively related in female non-contraceptive users (*p* < 0.001), and hormonal contraceptive users (*p* < 0.001), as shown in Figure 4(A). Similarly, average temperature and total testosterone showed a negative relationship in males (*p* < 0.001) and positive relationship in female non-contraceptive users (*p* < 0.001), and female hormonal contraceptive users (*p* < 0.001) as shown in Figure 4(B). Total-testosterone was also related to training week, where testosterone increased over the course of training (*p* < 0.001). As expected, males had higher levels of total testosterone than both female non-HC users and female hormonal contraceptive users (males: 459.88 ± 159.42, non-contraceptive users: 35.98 ± 11.64, female HC users: 33.90 ± 32.99 ng∙dL^−1^, both *p* < 0.001). Free testosterone was unrelated to maximal and average temperature (all *p* > 0.05) in all participants.

17β-estradiol was related to maximum daily temperature only in female non-contraceptive users (*p* = 0.025) such that increases in temperature were associated with decreases in 17β-estradiol concentration. The relationship between maximum temperature and 17β-estradiol in males did not reach statistical significance (*p* = 0.085). There was no relationship between 17β-estradiol and average daily temperature in any group. There was also no relationship between maximum or average daily temperature in female hormonal contraceptive users (*p* = 0.796, *p* = 0.593, respectively). Males and female hormonal contraceptive users had lower concentrations of 17β-estradiol than female non-contraceptive users (female non-contraceptive users: 67.31 ± 14.87, female hormonal contraceptive users: 27.82 ± 28.12, males: 26.70 ± 14.87 pg⋅mL^−1^, both *p* < 0.01) as shown in Figure 4(D).

SHBG was related to maximum daily temperatures in males only (*p* = 0.031) as shown in Figure 4(E). Average temperature had a significant relationship with SHBG in female non-contraceptive users only, as shown in Figure 4(F) (*p* = 0.010). The relationship between average temperature and SHBG did not reach statistical significance in males (*p* = 0.053). Males had lower SHBG than both female non-contraceptive users and female hormonal contraceptive users, and female HC users had higher SHBG concentrations than female non-users (males: 28.81 ± 11.89, female non-contraceptive users: 44.05 ± 22.50, female hormonal contraceptive users: 59.84 ± 55.59 nmol∙L^−1^, both *p* < 0.01). SHBG was also inversely associated with training, such that training duration was associated with increases in SHBG (*p* < 0.001).

There was no relationship between serum LH and daily maximum or average temperatures in any group, as shown in Figure 4(Gand H), respectively (*p* > 0.05). However, males showed lower serum levels of LH compared to both groups of females, and female non-contraceptive users had higher levels of LH than female hormonal contraceptive users (males: 4.21 ± 1.94, female non-contraceptive users: 6.27 ± 5.84, female hormonal contraceptive users: 5.78 ± 5.43 mIU∙mL^−1^ males vs. female non-contraceptive users and HC-users both *p* < 0.001 and female non-contraceptive users vs. female users: *p* = 0.035). LH also had a significant relationship with week of training (*p* = 0.030). Table 3 shows the results of the linear mixed‑effects model evaluating the relationship between maximum and average daily temperatures and serum total testosterone, estradiol, SHBG, and LH, respectively, from blood samples collected.

**Table 3.** Linear mixed-effects model results evaluating the relationship between maximum and average daily temperatures in total testosterone, estradiol, SHBG, and LH from intermittent blood samples in males (*n* = 112), female non-users (*n* = 62), and female contraceptive users (*n* = 63). (t-1) refers to the maximal or average daily temperature from the day before the hormonal measurement.

Panel A	Total Testosterone				Estradiol			
	*β ± SE*	*p*	*β ± SE*	*p*	*β ± SE*	*p*	*β ± SE*	*p*
Max Temp (t-1)	**-8.924 ± 1.627**	**0.000**			−0.834 ± 0.485	0.085		
Males	**0.000 ± 0.000**				0.000 ± 0.000			
Female non-contraceptive users	**-837.679 ± 46.998**	**0.000**			**90.224 ± 31.510**	**0.004**		
Female hormonal contraceptive users	**-842.327 ± 47.219**	**0.000**			−18.544 ± 19.517	0.342		
Male * max temp (t-1)	0.000 ± 0.000				0.000 ± 0.000			
Female non-contraceptive users * max temp (t-1)	**13.246 ± 1.569**	**0.000**			−1.635 ± 1.027	0.112		
Female hormonal contraceptive users * max temp (t-1)	**13.340 ± 1.561**	**0.000**			0.625 ± 0.690	0.365		
Week	**5.735 ± 1.075**	**0.000**			−0.584 ± 0.559	0.296		
Avg Temp (t-1)			**-8.860 ± 1.703**	**0.000**			−0.312 ± 0.426	0.464
Males			**0.000 ± 0.000**				0.000 ± 0.000	
Female non-contraceptive users			**-743.289 ± 43.570**	**0.000**			**66.759 ± 28.162**	**0.018**
Female hormonal contraceptive users			**-746.827 ± 42.735**	**0.000**			−23.205 ± 26.587	0.383
Male * avg temp (t-1)			**0.000 ± 0.000**				0.000 ± 0.000	
Female non-HC users * avg temp (t-1)			**12.269 ± 1.767**	**0.000**			−1.042 ± 1.121	0.353
Female HC users * avg temp (t-1)			**12.350 ± 1.700**	**0.000**			0.945 ± 1.131	0.404
Week			**5.723 ± 0.865**	**0.000**			−0.058 ± 0.508	0.908
Intercept	757.940 ± 54.090	0.000	709.061 ± 43.534	0.000	56.150 ± 17.925	0.002	36.118 ± 14.546	0.013
N	1,004		1,004		1,004		1,004	
	**SHBG**				**LH**			
**Panel B**	***β ± SE***	***p***	***β ± SE***	***p***	***β ± SE***	***p***	***β ± SE***	***p***
Max Temp (t-1)	**-0.290 ± 0.135**	**0.031**			0.002 ± 0.043	0.971		
Males	0.000 ± 0.000				0.000 ± 0.000			
Female non-contraceptive users	−3.488 ± 6.262	0.578			3.537 ± 2.965	0.233		
Female hormonal contraceptive users	12.107 ± 20.336	0.552			3.413 ± 3.480	0.327		
Male * max temp (t-1)	0.000 ± 0.000				0.000 ± 0.000			
Female non-contraceptive users * max temp (t-1)	**0.558 ± 0.211**	**0.008**			−0.052 ± 0.098	0.592		
Female hormonal contraceptive users * max temp (t-1)	0.788 ± 0.671	0.241			−0.072 ± 0.117	0.537		
Week	−0.666 ± 0.126	0.000			0.046 ± 0.045	0.305		
Avg Temp (t-1)			−0.239 ± 0.123	0.053			0.056 ± 0.036	0.118
Males			0.000 ± 0.000				0.000 ± 0.000	
Female non-HC users			−6.056 ± 5.481	0.269			2.948 ± 2.083	0.157
Female HC users			6.715 ± 18.231	0.713			1.320 ± 2.668	0.621
Male * avg temp (t-1)			0.000 ± 0.000				0.000 ± 0.000	
Female non-contraceptive users * avg temp (t-1)			**0.777 ± 0.227**	**0.001**			−0.040 ± 0.084	0.633
Female hormonal contraceptive users * avg temp (t-1)			1.166 ± 0.720	0.105			−0.004 ± 0.110	0.974
Week			**-0.595 ± 0.111**	**0.000**			**0.077 ± 0.036**	**0.030**
Intercept	45.489 ± 5.304	0.000	42.359 ± 4.333	0.000	3.754 ± 1.608	0.020	2.244 ± 1.137	0.048
N	1,004		1,004		994		994	

Each column contains results from a separate linear mixed effects model which controls for a fixed effect for location.

## Discussion

In this exploratory field-based analysis of men and women undergoing initial military training, higher ambient temperatures were associated with modest but consistent shifts in circulating reproductive hormones and related proteins, with sex-specific patterns. Higher temperature was often associated with lower hormone concentrations; this relationship was particularly prominent in estrogen-related measures (both urine and serum) in female non-contraceptive users and in total testosterone in males. Taken together, our findings suggest that ambient temperature is associated with reproductive hormone profiles during prolonged physical training, independent of training duration *per se*.

We observed these relationships within a training environment that includes sustained physical workloads and multiple concurrent stressors known to influence reproductive endocrine function. Using this same cohort, we previously reported that 75% of non-hormonal contraception users in this group had ovulatory suppression during initial military training [25]. The present analysis cannot distinguish the independent effects of heat exposure from other training-related stressors. However, since the temperature relationships were distinct from the relationships with time in training, we suggest that ambient temperature may represent an independent environmental contributor to endocrine variability during military training.

### Estrogen, follicle stimulating hormone, and luteinizing hormone

The most consistent relationships between ambient temperature and hormone levels were observed for estrogen-related measures in female non-contraceptive users. Urinary E1C, FSH, and serum 17-β estradiol levels were inversely associated with ambient temperature, suggesting coordinated sensitivity within the estrogen feedback axis [37]. These findings align with animal models demonstrating that heat stress impairs estrogen synthesis [8,10,38] and disrupts the estrous cycle [39,40] which can lead to decreased fertility [41,42] and reproductive function [42]. However, extrapolation from controlled animal models to free-living humans should be made cautiously. The magnitude of the observed estradiol reductions in the present analysis was modest relative to the full range of concentrations observed across individuals and menstrual phases, but even modest downward shifts might be biologically meaningful when occurring repeatedly during prolonged training. In contrast, serum estradiol was not associated with ambient temperature in hormonal contraceptive users or men. This pattern may reflect reduced physiologic variability in estradiol among hormonal contraceptive users due to exogenous hormone regulation, and/or lower absolute estradiol concentrations in both hormonal contraceptive users and men that could limit sensitivity for detecting modest temperature effects. Together, these findings suggest that temperature-related variation in circulating estrogen may be most detectable in women with no exogenous hormone exposure.

Beyond reproductive function, estrogen plays a central role in acute human thermoregulatory responses during heat stress [43–45]. Specifically, estrogen has both reflex and local vasodilator influences, which can augment heat dissipation by increasing cutaneous blood flow [46–49]. If elevated ambient temperatures are associated with lower circulating estrogen, this could diminish vasodilatory capacity and contribute to thermal strain, although this mechanism could not be evaluated in the present analysis. Notably, neither urinary LH nor PDG was associated with ambient temperature, which is not surprising given the low percentage of volunteers that had evidence of luteal activity [25]. Likewise, serum LH was not temperature-associated in any group. While animal studies have reported heat-induced suppression of gonadotropin signaling [38,50,51], these models involve sustained or experimentally controlled heat stress, suggesting the magnitude or duration of heat exposure may be important determinants of gonadotropin responsiveness [52]. Research has commonly cited the influence of female sex hormones on thermoregulatory responses, primarily under laboratory controlled conditions with the influence of menstrual cycle and contraceptive use evaluated [45,53–55], but it is possible that the relationship is bidirectional, with a larger impact of ambient heat stress on hormonal concentrations, as previous literature has shown the environment is more impactful on thermoregulatory responses than sex hormone concentration [56].

### Testosterone

Total testosterone demonstrated clear sex-specific associations with ambient temperatures. In males, higher maximum and average daily temperatures were associated with lower circulating total testosterone. These findings are consistent with previous animal reports showing testicular hyperthermia suppresses testosterone concentrations [57–59], with partial recovery following heat acclimatization [60]. The magnitude of the decrease in testosterone in males in the present analysis was moderate, corresponding to an approximate 18% decrease from males with the highest testosterone concentrations (178ng⋅dL^−^1 decrease over testosterone range 110–965 ng⋅dL^−1^). For individuals who may have borderline low androgen status at baseline, such decreases may contribute to negative consequences; however, in otherwise healthy individuals this moderate decrease is unlikely to be consequential.

In contrast, in both female non-contraceptive users and hormonal contraceptive users, higher temperatures were associated with modest increases in total testosterone. The positive association between ambient temperature and total testosterone observed in females was unexpected and is not well characterized in the literature. The influence of environmental heat exposure on circulating androgens in women has been minimally studied. Mechanistic explanations for this pattern are currently lacking but could be related to a negative feedback relationship between estradiol-related systems and testosterone (ie, since estradiol was decreasing, testosterone was able to increase). Testosterone has important roles in musculoskeletal and metabolic health in women, but variables associated with these relationships were beyond the scope of this analysis. Importantly, the present findings should be interpreted as observational and hypothesis-generating rather than evidence of a defined physiological response.

Total testosterone increased across weeks of training in all groups, which is consistent with previous reports of training-associated endocrine adaptation [61–63]. These training-related increases do not explain the temperature associations, which persisted after accounting for training duration. In males, the absence of an association between ambient temperature and serum LH limits inferences regarding the level of regulation affected, as the present data cannot distinguish between central alterations in hypothalamic-pituitary signaling and peripheral effects on testicular steroidogenesis. Serum FSH was not measured, further constraining mechanistic interpretation.

### Sex hormone binding globulin

Regarding protein responses, SHBG demonstrated a positive relationship with maximum and average temperatures in males only. The basis for this sex-specific pattern is unclear. In females, no association between ambient temperature and SHBG was observed in either non-contraceptive users or hormonal contraceptive users. Estrogen is a well-established regulator of hepatic SHBG production [64,65], and altered SHBG concentrations have been reported in clinical populations characterized by disrupted sex steroid balance such as polycystic ovary syndrome [66]. However, in the present observational analysis, we could not evaluate whether temperature-associated variation in SHBG reflects changes in hepatic production, alterations in sex steroid availability, or other regulatory pathways. Overall, our findings suggest that ambient temperature may influence SHBG regulation in males, but the physiological significance and mechanisms underlying this association remain unclear and warrant further investigation.

### Limitations

First, only females who were not utilizing any form of hormonal contraception provided daily urine samples and thus are the only group able to be compared in that manner, with menstrual cycle phase not controlled for or evaluated. Additionally, over the course of initial military training, compliance varied in daily urine samples, as individuals did not always provide daily samples over the 9+ weeks, as expected in a field setting. However, future work over a shorter timeframe could investigate daily samples with greater temporal resolution and the potential influence of environmental temperatures. Third, this investigation was limited to one initial military training, class that took place from August to October 2021, whereby all individuals experienced the same environmental changes. Future investigations could expand the timeframe and weather conditions such that there are larger ranges in temperature captured within the sample. Additionally, heat stress is not restricted to only ambient temperature changes. Humidity (both absolute and relative) plays an important role in thermoregulation and heat stress, but humidity data were not available for this analysis. Individual level heat strain and core temperature were also not measured in this analysis, making inference regarding physiological heat stress, or the relationship between body temperature and sex hormone concentration impossible. Another limitation of this analysis lies with body weight, as variations in body weight can affect hormonal concentrations, particularly in females. While body weight was measured at baseline, it was not utilized in the model as changes in body weight likely varied over the course of the 9 weeks. Finally, as a secondary analysis, this investigation relied on the measurement of circulating sex hormones, which are one aspect of reproductive function but cannot alone indicate detriments in reproductive health [67,68]. Future research should include additional measures of reproductive function in women and men to comprehensively evaluate the potential impact of ambient temperature on reproductive health in active individuals.

## Conclusion

Ambient temperature was associated with small but consistent, sex-specific differences in reproductive hormones during initial military training. Higher temperatures were linked to lower estrogen-related measures in women not using hormonal contraception and to lower total testosterone in men, while higher temperatures were associated with modest increases in total testosterone in women. Although these associations were observed within a complex training environment and were modest in magnitude, they highlight ambient temperature as a potential contributor to endocrine variability during prolonged physical training and underscore the need for future studies designed to isolate heat exposure from other training-related stressors.

## Abbreviations

LH: luteinizing hormone
E1C: estrone conjugates
PDG: pregnanediol glucuronide
FSH: follicle stimulating hormone
BMI: body mass index
NOAA: National Oceanic and Atmospheric Administration
